# A Rare Subtype in an Uncommon Location: Multimodal Radiologic and Histopathologic Evaluation of a Solid Variant Aneurysmal Bone Cyst in the Radial Head

**DOI:** 10.5334/jbsr.4012

**Published:** 2025-07-04

**Authors:** Furkan Özdem, Gözde Elif Taşar Kapakli, Rasime Pelin Kavak

**Affiliations:** 1 Health Sciences University Ankara Etlik City Hospital, Department of Radiology, Ankara, Turkey; 2 Health Sciences University Ankara Etlik City Hospital, Department of Pathology, Ankara, Turkey

**Keywords:** aneurysmal bone cyst, solid variant, radial head, radiology, histopathology

## Abstract

A 30-year-old male presented with pain and swelling in the left elbow. Multimodal radiologic evaluation revealed an expansile, lytic bone lesion with fluid-fluid levels and surrounding soft tissue edema, localized in the radial head. Histological examination confirmed the diagnosis of a solid variant of aneurysmal bone cyst (SVABC), an anatomically rare site for such lesions. The lesion was managed with curettage, leading to complete clinical resolution.

*Teaching point:* integrating radiologic and histologic findings to an accurate diagnosis and effective management of SVABC, especially when occurring in atypical locations where they may mimic more aggressive neoplasms.

## Introduction

Aneurysmal bone cyst (ABC) is a rare cystic and expansile benign bone lesion that accounts for 1% of all bone tumors [1, 2]. The solid variant of aneurysmal bone cyst (SVABC) is a very rare subtype of ABC, comprising approximately 3.4%–7.5% of all ABCs. This variant is distinguished by a predominance of solid components, rather than the typical hemorrhagic cystic spaces observed in conventional ABCs [3].

An SVABC localized in the radial head is presented, with detailed multimodal imaging and histological correlation.

## Case Report

**Figure 1:**
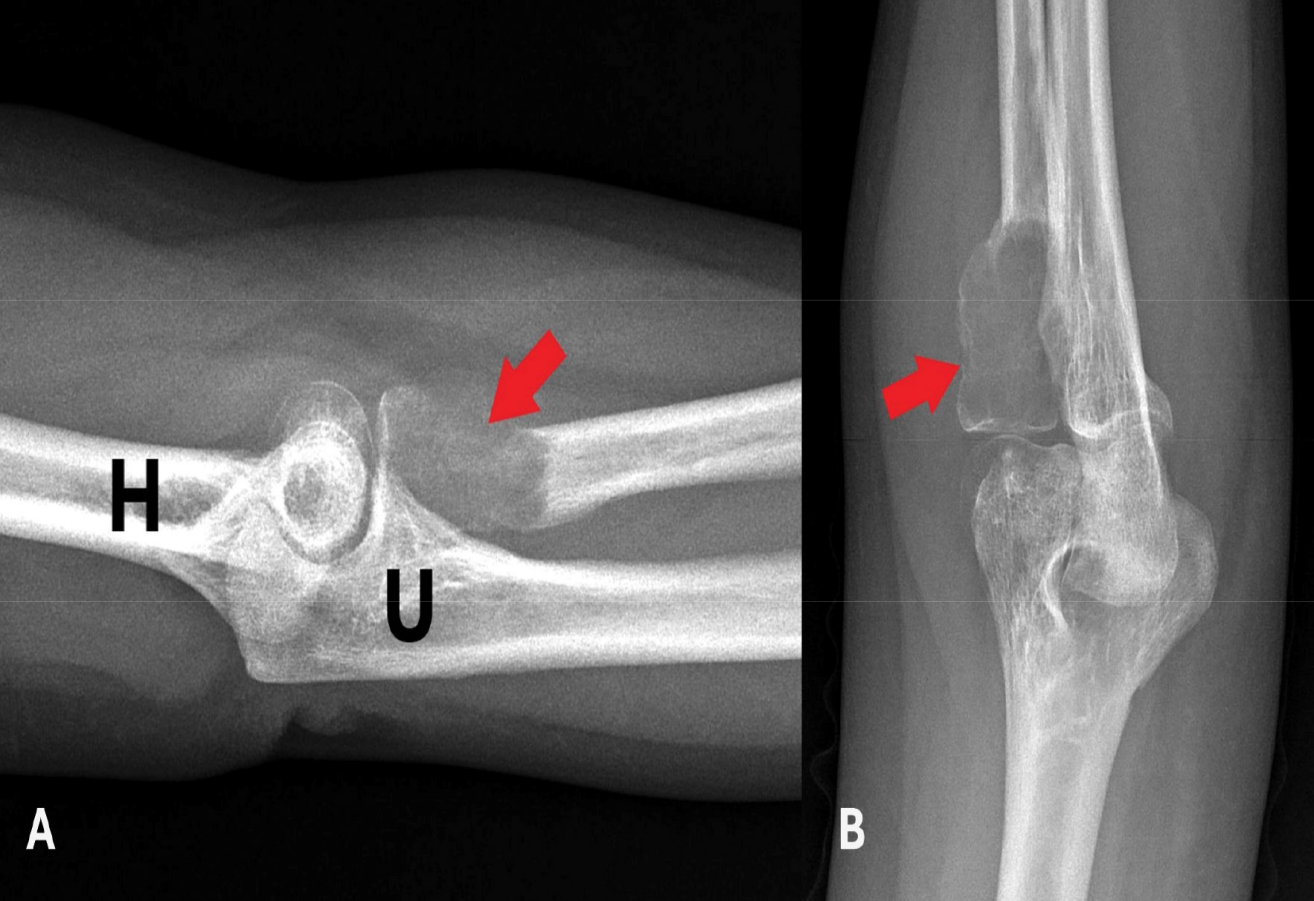
**A‑B** On plain radiography, a well‑defined, expansile, lytic bone lesion with thin internal septations is present in the radial head (indicated by red arrows). Adjacent osseous structures are labeled as humerus (H) and ulna (U).

**Figure 2:**
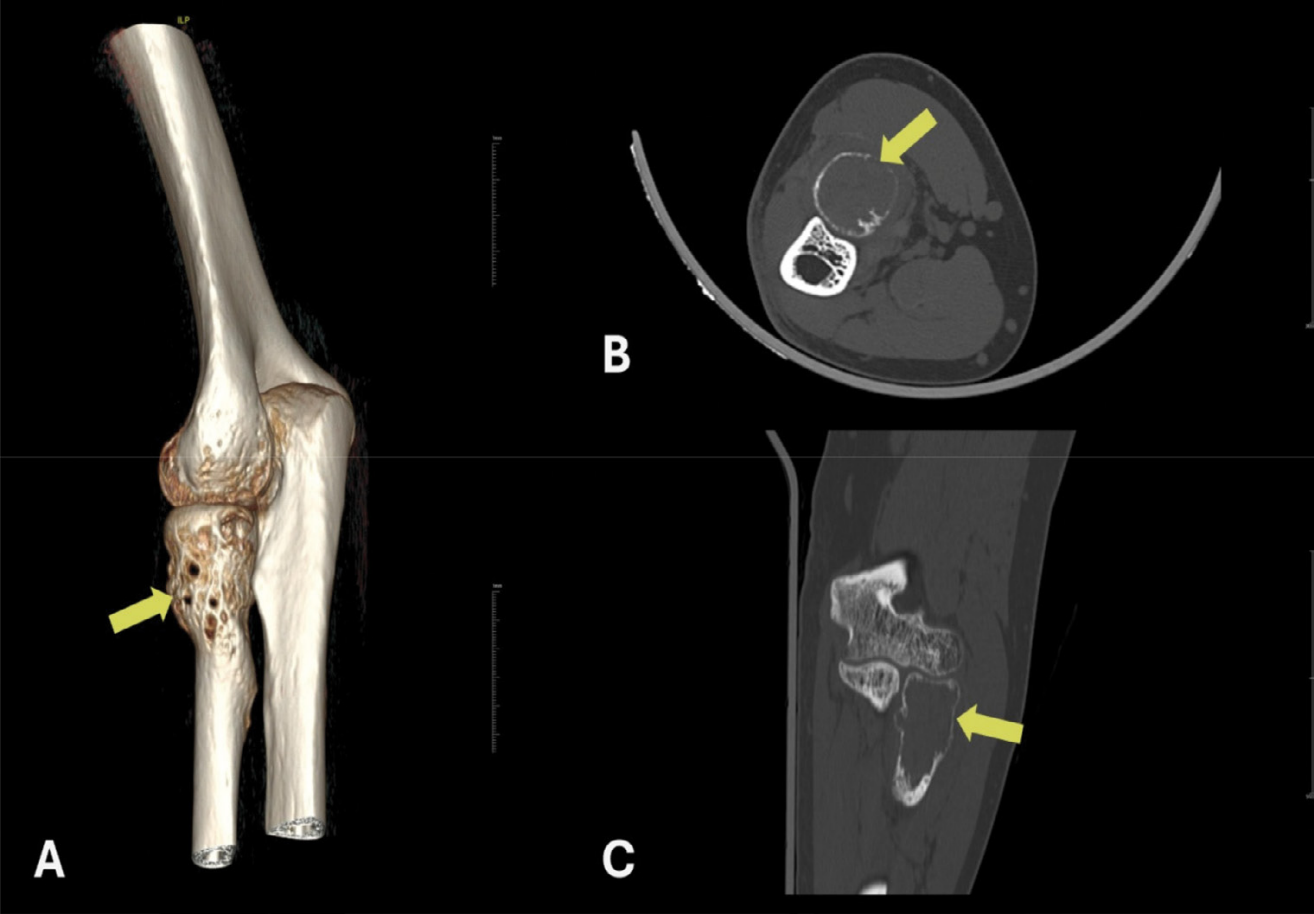
Three‑dimensional reformatting **(A)**, axial **(B)**, and coronal **(C)** CT images demonstrate a well‑defined, lytic and expansile bone lesion located at the proximal metadiaphyseal region of the radius. The lesion is associated with cortical thinning (indicated by yellow arrows).

**Figure 3:**
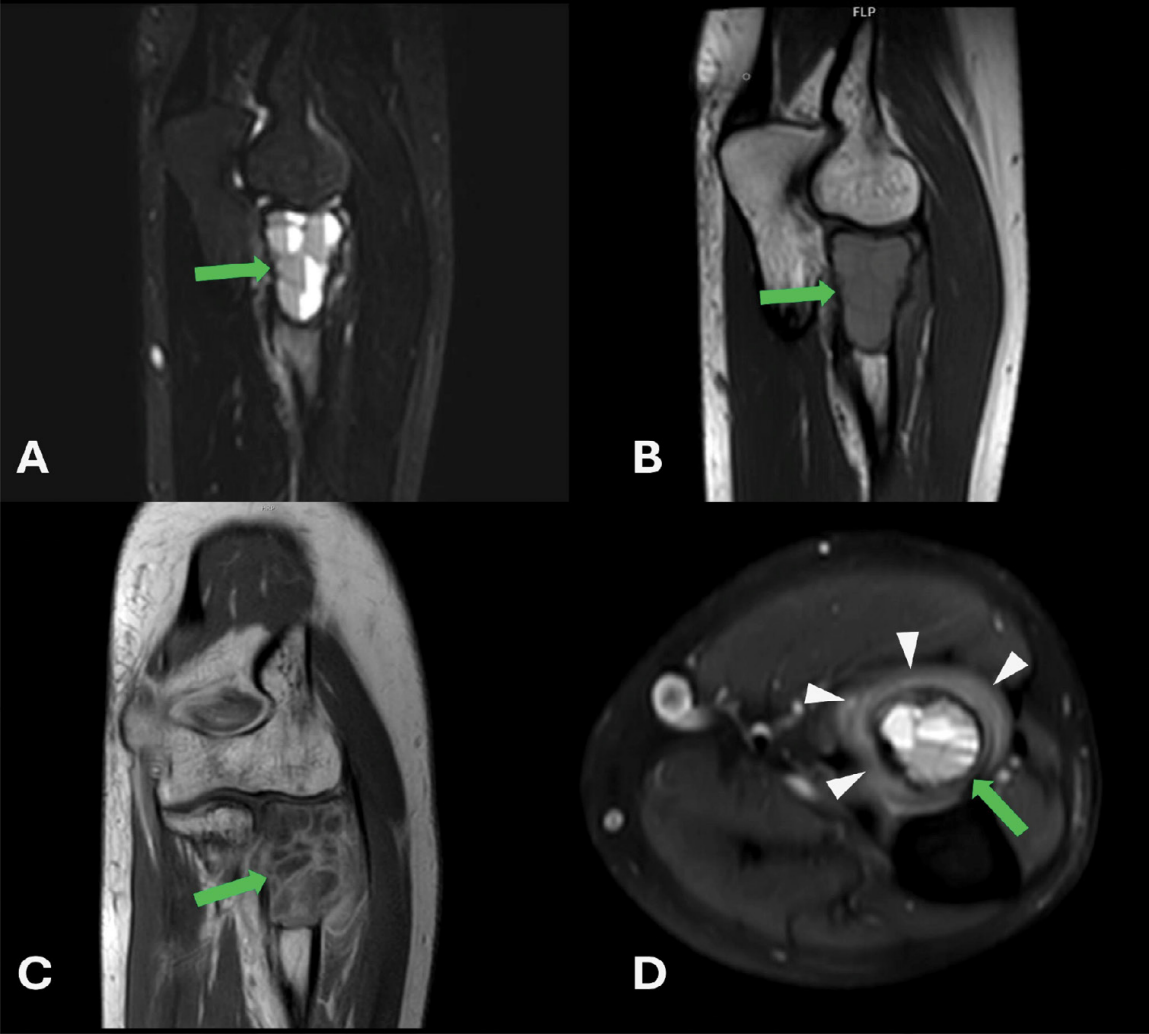
**A‑D** MRI examination of the left elbow. **(A)** On the STIR sequence, the lesion demonstrates heterogeneous hyperintense signal characteristics and fluid levels. **(B)** On T1‑weighted images, it appears mildly hyperintense compared to adjacent muscle tissue. **(C)** Post‑contrast T1‑weighted images show peripheral and septal enhancement (green arrow). Internal fluid‑fluid levels are observed, supporting a multiloculated cystic architecture (green arrow in D). The lesion causes expansion of the bone without associated cortical erosion and destruction. **(D)** Axial proton‑density (PD)‑weighted images demonstrate increased signal intensity in the adjacent soft tissues and muscle planes, consistent with edema (white arrowheads).

**Figure 4:**
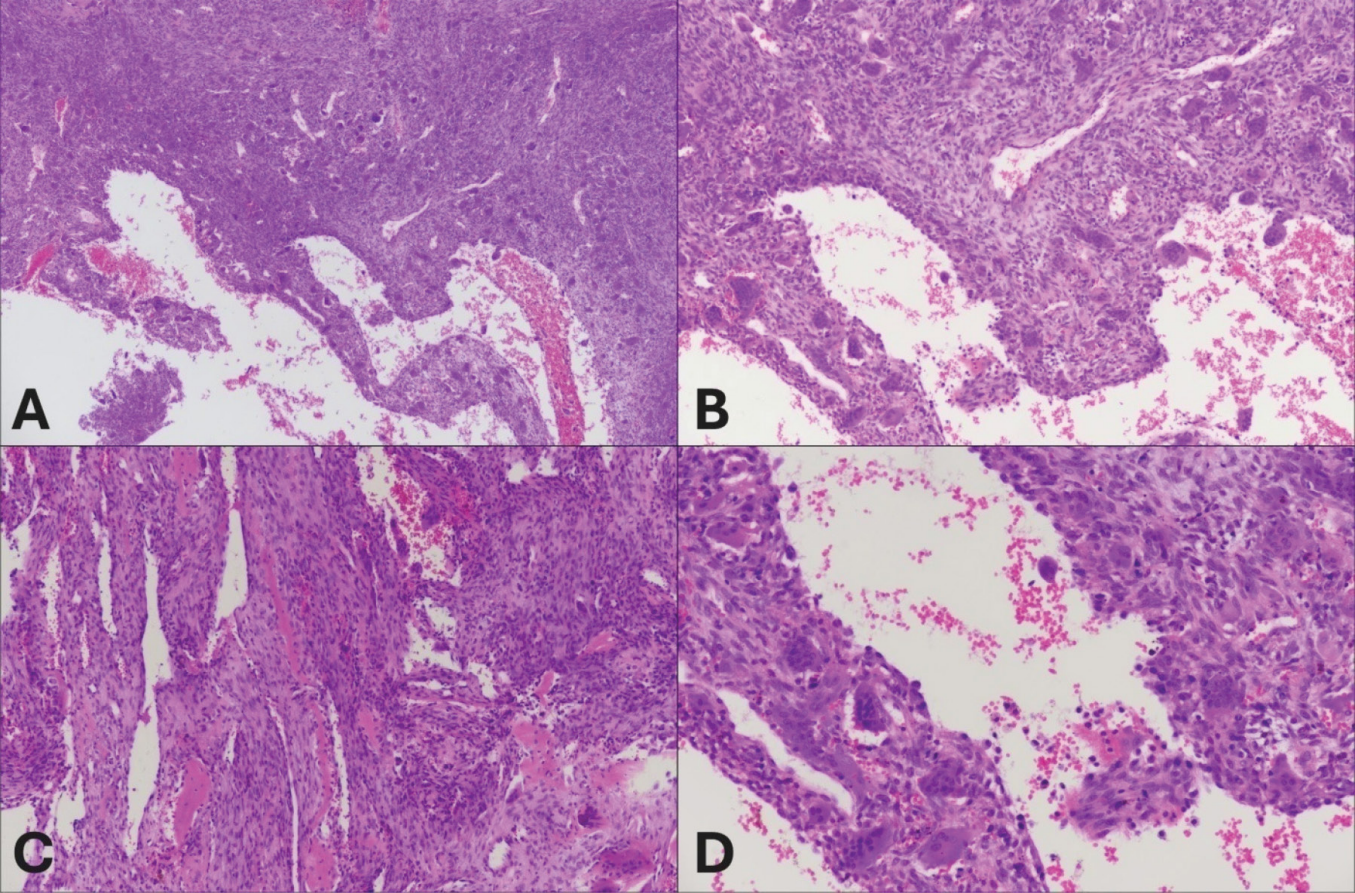
**A‑D** Histological features of the solid variant of aneurysmal bone cyst (SVABC). **(A)** Solid areas are more prevalent than cystic cavities. There are osteoclastic giant cells and blood filled cystic spaces (H&E, × 100). **(B)** Mononuclear neoplastic cells with numerous giant cells. There is no atypia (H&E, × 100). **(C)** Blood‑filled slit‑shaped cystic spaces separated by cellular septa containing fibroblasts, giant cells, and woven bone (H&E, × 200). **(D)** Numerous giant cells in connective tissue line large sinusoidal spaces (H&E, × 200).

A 30‑year‑old male patient presented with complaints of pain and mild swelling in the left elbow region. He had no history of trauma or surgery, no chronic illnesses, nor medication use. Laboratory investigations revealed no pathological findings. A well‑defined expansile lytic radiolucent bone lesion with thin internal septations was seen in the proximal radius on radiographs [Figure 1A‑B]. Computed tomography (CT) with three‑dimensional volume‑rendered images revealed a lytic bone lesion located in the proximal metadiaphyseal region of the radius, causing cortical thinning and osseous expansion [Figure 2]. Magnetic resonance imaging (MRI) confirmed the heterogeneous multicystic lytic bone lesion located with hyperintense signal characteristics relative to adjacent muscle tissue on both T1‑ and T2‑weighted sequences, and with some fluid‑fluid levels. Contrast images showed enhancement of the lesion walls and the internal septations [Figure 3A‑C]. Increased signal intensity consistent with edema was noted in the adjacent soft tissues and muscles [Figure 3D]. Although the imaging features were indicative of an ABC, the presence of perilesional soft tissue edema raised concern for a more aggressive or potentially malignant lesion. Histological evaluation of a biopsy specimen revealed features consistent with an SVABC [Figure 4A‑D]. Surgical treatment with curettage was performed. The pain and swelling of the left elbow completely resolved.

## Discussion

SVABC was first described by Sanerkin et al. in 1983 [4]. SVABC is a rare subtype of aneurysmal bone cyst, more frequently observed in young individuals and female patients [5]. This lesion typically tends to involve the axial skeleton and short tubular bones [3, 6, 7]. The differential diagnosis of SVABC includes telangiectatic osteosarcoma, giant cell tumor, Ewing sarcoma, and Langerhans cell histiocytosis [5, 6].

SVABC can resemble malignant bone tumors on imaging, which may lead to diagnostic errors [4, 6]. Radiologically, SVABC lesions typically present as well‑defined, expansile and lytic, often with internal fluid‑fluid levels, similar to those observed in conventional ABC [5]. However, certain features, such as perilesional edema, solid components extending into adjacent soft tissues, and erosive or destructive bone alterations may also be present, further contributing to their resemblance to malignant lesions. Therefore, histological evaluation remains crucial for final diagnosis and for guiding appropriate surgical and clinical management [5, 6].

Histological examination of the SVABC may reveal areas of osteoclastic giant cell proliferation and solid‑cystic components that can resemble those of a malignant giant cell tumor. Hemorrhagic cystic spaces are observed in the majority of lesions and are often predominant within the solid components. Fibroblastic proliferation is typically observed within the solid areas. The absence of pleomorphic cells and malignant osteoid formation excludes the diagnosis of osteosarcoma [4, 5].

## Conclusion

SVABC is a rare benign bone lesion that may mimic malignant tumors both radiologically and histologically, leading to potential misdiagnosis and overtreatment. This case illustrates the significance of comprehensive multimodal imaging and histological evaluation in establishing an accurate diagnosis of SVABC, particularly when the lesion arises in atypical locations such as the radial head. Awareness of this rare entity and its potentially distinguishing features contributes to appropriate surgical planning and avoiding unnecessary aggressive treatments.
